# Amplifying the Voice of the Patient in Clinical Research: Development of Toolkits for Use in Designing and Conducting Patient-Centered Clinical Studies

**DOI:** 10.1007/s43441-020-00176-6

**Published:** 2020-07-02

**Authors:** TransCelerate Biopharma Toolkits Core Team, Mary Elmer, Cathy Florek, Lori Gabryelski, Alison Greene, Anne Marie Inglis, Karen L. Johnson, Tanja Keiper, Sean Ludlam, T. J. Sharpe, Kathyjo Shay, Fabian Somers, Catherine Sutherland, Michele Teufel, Stephen Yates

**Affiliations:** 1 Merck & Co., Inc., Upper Gwynedd, PA 19446 USA; 2 Bristol Myers Squibb, Princeton, NJ 08540 USA; 3 Patient-Centered Outcomes Research, Genentech, San Francisco, CA 94080 USA; 4 Clinical Operations, GlaxoSmithKline, 1250 South Collegeville Road, MS UP 4210, Collegeville, PA 19426 USA; 5 Global Clinical Operations, EMD Serono/Merck Healthcare KGaA, 64293 Darmstadt, Germany; 6 Clinical Records Management, Allergan, Madison, NJ 07940 USA; 7 Patient Advisor, Starfish Harbor, Fort Lauderdale, FL 33316 USA; 8 Clinical Science Center of Excellence, Astellas Pharma Global Development, Inc., Northbrook, IL 60062 USA; 9 Clinical Development, UCB Pharma, Brussels, Belgium; 10 Drug Development Operations, Allergan, Buckinghamshire, SL7 1YL UK; 11 Development Operations, AstraZeneca, Wilmington, DE 19850-5437 USA; 12 Global Clinical Development, UCB Pharma, Raleigh, NC 27617 USA

**Keywords:** TransCelerate Biopharma Inc., Patient engagement, Clinical research, Clinical trial, Patient advocacy, Protocol, Patient-focused, Participant

## Abstract

Incorporating patient perspectives into clinical studies is recognized as important to the development of high-quality, safe, and effective fit-for-patient medicines. However, no widely accepted methodology to help design more patient-centered studies has been established systematically. TransCelerate Biopharma Inc., a non-profit organization promoting collaboration across biopharmaceutical companies, organized a Patient Experience (PE) Initiative to create tools to intentionally include the patient perspective into the design and implementation of clinical studies. The resulting tools include the Patient Protocol Engagement Toolkit (P-PET), to engage patients early in protocol development, and the Study Participant Feedback Questionnaire (SPFQ), to assess patient experiences during clinical studies. To develop these toolkits, TransCelerate conducted a literature review and identified aspects of clinical studies that patients find either valuable or burdensome, or that affect participation, adherence, and engagement in a clinical study. The concepts identified were refined through elicitation of feedback from patient advisors, clinical study site advisors, and subject matter experts from member companies (MCs) of TransCelerate. This feedback was considered in identifying gaps, defining scientific methodology to understand how to evaluate patients’ needs, and developing and refining the P-PET and the SPFQ. As part of the development process, descriptions/drafts of the tools were shared with patients, clinical site advisory groups, MCs, and the US Food and Drug Administration, and then revised. MCs simulated use of the tools, and feedback was incorporated into the final versions of the P-PET and SPFQ prior to public release. The P-PET and SPFQ are available free on the TransCelerate website.

## Background

The importance of incorporating patient perspective into the development and execution of clinical studies is increasingly recognized, demonstrated by the broad scope of initiatives and regulatory frameworks and guidances dedicated to making clinical studies less burdensome for patients. Sponsors conducting clinical studies acknowledge the significance of patient input, but there is currently no widely accepted, vetted methodology established to obtain this input. To maximize the inclusion of patient perspective in clinical studies, the TransCelerate PE Initiative has endeavored to further understand the impact of the patient experience in clinical study design and to develop tools to support the development and conduct of patient-centered clinical studies across clinical development.

The PE team recognized the need to create tools to:

- support engaging patients early in clinical protocol development, and
- assess patient experience throughout the conduct of a clinical study from enrolled study participants.

The resulting tools, the Patient Protocol Engagement Toolkit (P-PET) and the Study Participant Feedback Questionnaire (SPFQ) Toolkit, are described herein.

The P-PET provides considerations for how to effectively engage patients in gathering meaningful patient feedback during protocol development and includes the following:

- *A User Guide* that supports those involved in developing clinical research studies in (1) understanding the value of implementing the P-PET in clinical studies, (2) understanding how to leverage and implement the P-PET in clinical study design, and share best practices on having meaningful discussions with patients, (3) socializing the toolkits with study sponsors and other key stakeholders to seek support as needed, (4) providing example case studies.
- *A Resource Guide* that includes a set of sample questions for consideration during an engagement with patients. Example visual aids are provided to facilitate clear communication of study design and protocol-related concepts.
- *Templates* to support engagements with patients and provide feedback to study teams and patients.

The SPFQ is a toolkit for gathering patient feedback while participating in a clinical study. It includes.

- *the SPFQ Implementation User Guide*, to support clinical researchers in the customization and implementation of the SPFQ,
- *the SPFQ Socialization Presentation*, for initial sponsor discussions, and
- *the SPFQ*, a set of three brief patient questionnaires designed to capture patient study experience at the beginning, during, and end of each study, independent of disease and treatment.

## Methods

A phased approach to the development of the PE toolkits was implemented.

Key concepts in clinical study participation were identified in the literature and by engaging stakeholders, particularly patients. A literature review was conducted, identifying aspects of clinical studies that patients consider valuable or burdensome or that could affect participation, protocol adherence, and engagement. The concepts identified were used to develop a framework for understanding what was important to patients. To assess the framework and elicit experience information, key stakeholders were interviewed, including patients (via Patient Advisory Board [PAB] and patient interviews), research/clinical study sites (via Site Advisory Group [SAG], composed of representatives from clinical study sites), and interviews with subject matter experts (SMEs) from TransCelerate MCs. Specifics are described in the results section under Part 1.

As a result, the PE team established the need to create tools to support engaging patients early in clinical protocol development and to assess study participation experience throughout the conduct of a clinical study from enrolled participants. Relevant findings from the literature review and stakeholder inputs were used to develop draft toolkits suitable for assessing face- and content-validity. The initial draft toolkits were refined in an iterative process by further engagement with stakeholders, including PABs, SAGs, a health authority, and SMEs from MCs. Specifics are described in the results section under Part 2. Relevant feedback was considered in developing and refining the toolkits to ensure all aspects of patient experience were covered.

Finally, use of the toolkits was simulated by MCs and comments were addressed in the toolkits before finalization for public release.

**Fig. 1:**
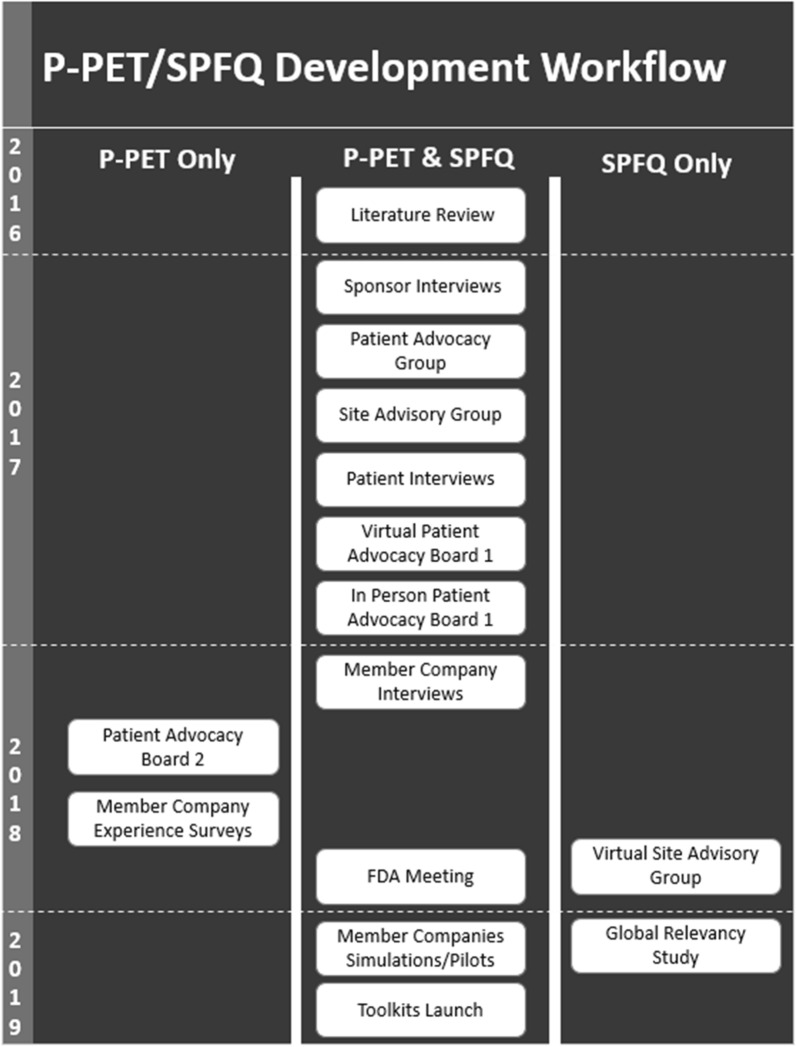
P-PET/SPFQ toolkit development timeline

The timeline for the development of the P-PET and SPFQ Toolkit is depicted in Fig. 1.

### Part 1: Shared Research: Literature Review and Stakeholder Research

#### Part 1a: Literature Review and Framework for Initial Development

The PE team conducted a literature search focused on peer-reviewed publications, white papers, presentations, and abstracts in conference proceedings in the public domain, published primarily after 2012. The search was for publications that asked (or answered) the following questions:

1. What do patients perceive as burdensome about their clinical study experience?
2. What do patients perceive as valuable about their clinical study experience?
3. What drives patient adherence?
4. What increases patients’ willingness to participate in a clinical study?
5. What activates patients in their clinical study journey?

The identified literature was reviewed, summarized, and categorized by the PE team based on the following topics related to patient perspective regarding clinical studies:

- Patient *burden*
- *Value* of participation
- *Willingness to participate*
- *Activation/empowerment* of participants
- *Adherence* to clinical study medication and procedures

Consistent themes were identified and used to develop a framework of core concepts, themes, and potential topics about which patient perspective and insights could be solicited.

**Fig. 2:**
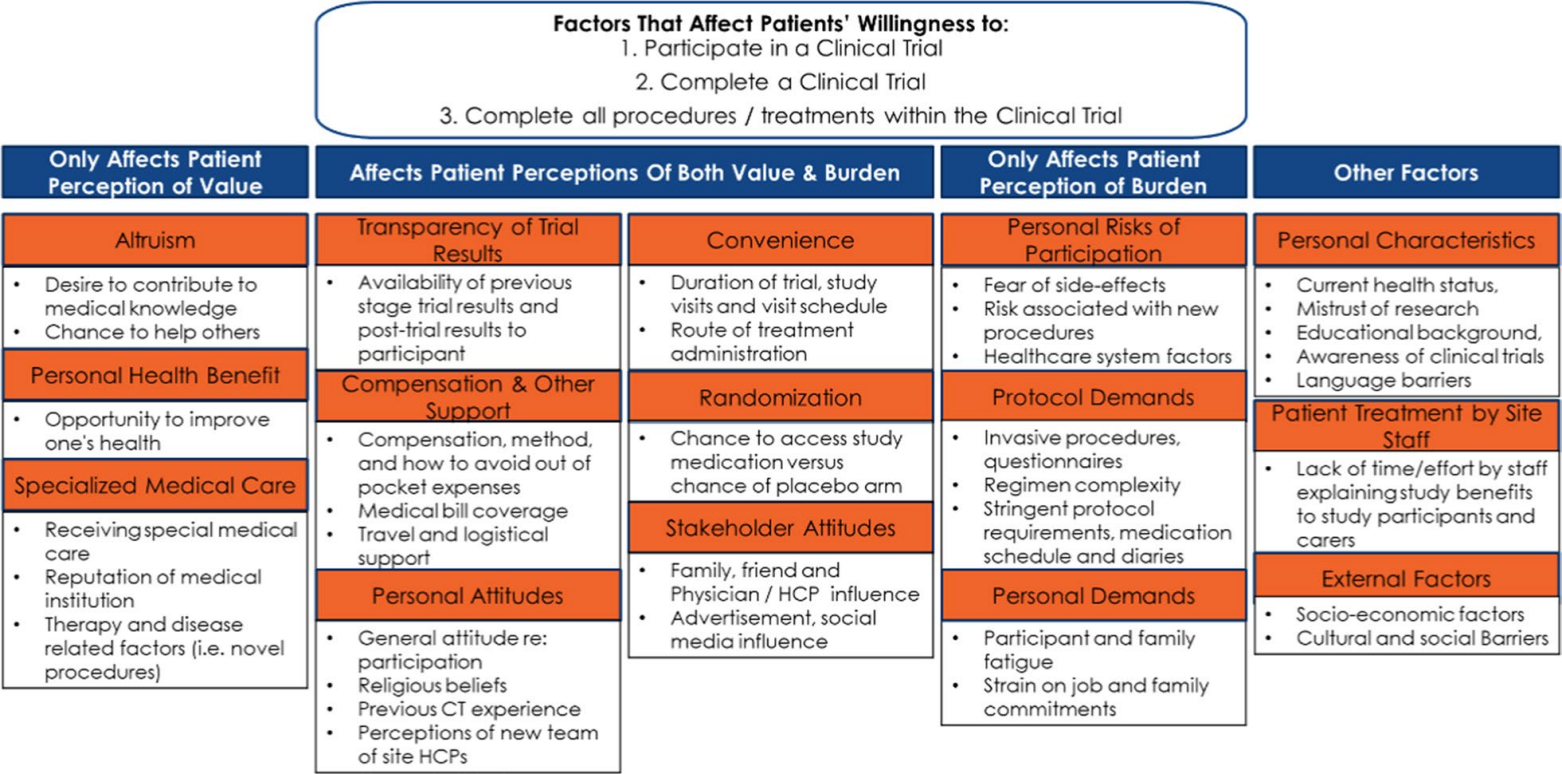
Framework for factors that affect patient perceptions of clinical studies

The concepts identified were used to inform a participation framework. Burden and value were determined to be at the root of the decision to participate and remain enrolled in a clinical study. The framework was developed by categorizing factors that affect (Fig. 2).

1. patient perception of value,
2. patient perceptions of both value and burden
3. patient perception of burden, and
4. other factors.

The PE team then considered how questions of value and burden could be organized in the context of the protocol design process. TransCelerate’s Common Protocol Template (CPT) is a protocol template with common elements and structure, suitable for adoption across the industry. The PE team used the CPT to identify elements of a study protocol about which patients’ feedback would be valuable. The elements were grouped into discussion categories and formed the basis for further research with patients; a discussion guide and questions for use with a PAB, and a patient interview script for 1:1 patient interviews were developed (Part 1b).

Further background on the patient journey through the clinical study process (identifying, evaluating, and enrolling in a clinical study, participation in the study, and the post-study experience) was also gathered from the literature for patients who had participated in Phase 2–4 clinical studies. This included identifying what sources of information about clinical studies are available to patients; how patients may find a participating site for a particular study; patient considerations when deciding whether to take part in a clinical study; and input from family/friends/caregivers.

#### Part 1b: Stakeholder Research

Stakeholders involved in patient engagement activities and protocol development/execution were interviewed to assess the framework, concept questions, and to elicit experience information. Key stakeholders initially included patients (the PAB), clinical study sites, and MCs. The goal was to gain a better and broader understanding of face- and content-validity and reliability of the identified factors in the framework.

##### PAB I (First Meeting)

The first face-to-face PAB was held with 10 patient advisors with chronic conditions from North America, Europe, and Australia/South America. The CPT elements were reviewed to establish a systematic methodology for how a sponsor might consider conducting PABs to receive relevant feedback about a planned protocol prior to protocol finalization. Patients were given elements of a clinical study to review, according to section of the CPT, as follows: introduction/background, treatment information, overall study design, safety and procedures, schedule of activities, lifestyle, study results, and post-study considerations.

The goal was to gain an initial appreciation of what factors impacted patients (positively or negatively) in a clinical study and on which elements patients would prefer to be consulted.

The PAB also reviewed an initial draft of an interview script used in the development of the SPFQ.

##### SAG I

The objective of meeting with a SAG was to provide an overview of the TransCelerate PE Initiative and to assess (1) factors that can have a positive impact on patient participation, (2) burden at the site level that may negatively impact patient experience, and (3) how sponsors can address that burden to create a more positive experience. A virtual SAG meeting was conducted with 10 representatives from sites in North America, Europe, and South America. Multiple roles were represented among the SAG members (including Clinical Operations Director, Investigator, Medical Director, SVP of Operations, Pharmacist, Budget/Contract Representative and Clinical Research Coordinator, Supervisor, Administration Manager).

The SAG members also commented on concepts identified by the PE team from the literature search (Fig. 2). They ranked factors perceived to most affect patients’ perception of value and burden with regard to participating in, adhering to, and completing a clinical study.

##### Patient Interviews

Individual qualitative patient interviews were conducted to identify concepts of importance to patients who had participated in Phase 2–4 clinical studies. Trained interviewers from a third-party vendor used Institutional Review Board (IRB)-approved open-ended interview materials based on a modified patient-reported outcomes (PRO) development methodology (US FDA Guidance for Industry: Patient-reported Outcome Measures: Use in Medical Product Development to Support Labeling Claims, December 2009; EMA Reflection Paper on the Regulatory Guidance for the Use of Health-related Quality of Life [HRQL] Measures in the Evaluation of Medicinal Products, July 2005) to conduct 1 hour videoconference interviews.

Nineteen patients of diverse ages, races, and disease backgrounds from the US and Europe who had participated in randomized, controlled clinical studies conducted in the US and United Kingdom were interviewed. Patient inclusion criteria included participation in a randomized clinical study (Phase 2 or later) within past 2–3 years, ≥ 18 years of age, and consent to participate in the interview.

The PE team reviewed the interview transcripts and identified common themes and concepts from patients, and then categorized them into higher level concepts.

##### MC Interviews

Interviews with 48 subject matter experts (SMEs) from 12 TransCelerate MCs were conducted to understand what topics sponsor organizations felt were important for effective patient engagement. The SMEs were decision-makers regarding study design and operational plans from various functions, levels, and diverse therapeutic areas. They were asked about the current level and means of patient engagement, and the potential value and use of proposed patient experience toolkits.

As with the PAB, elements of the CPT were used as a guide for interview discussion topics and for protocol elements that could impact patient experience in a clinical study. Specifically, protocol elements were assessed for impact in terms of its potential to affect willingness to participate (low to high) and difficulty to modify (low to high).

The PE team interviewed SMEs within their own MCs using a questionnaire based on the following themes:

- Respondent profile to ensure coverage of key stakeholders and therapy areas
- Existing level of patient engagement within the MC
- Current modes of patient engagement within the MC
- Perceived value and challenges of engaging patients in protocol development
- Potential modes and scenarios for P-PET use
- Drivers and barriers to P-PET implementation
- Impact vs. difficulty of modifying protocol elements based on patient input
- Feedback on the patient experience concepts (Fig. 2) regarding
  - Content
  - Organization
  - Relative impact
  - Ability to modify future clinical studies

Responses were anonymized and aggregated.

This feedback on the conceptual framework was considered for incorporation into the draft P-PET and in the development of the initial drafts of the SPFQ.

### Part 2: Development of Draft Toolkits

#### Part 2a: P-PET Development

The initial draft of the P-PET was based on concepts from the literature review, inputs from the initial PAB, SAG, patient interviews, and MC SME interviews. A process map and a prototype tool that housed questions believed to be relevant in eliciting feedback from patients was developed. These questions became the basis for the P-PET Resource Guide. A patient survey was built to gauge the protocol elements about which patient advisors should be consulted, and a first draft of the P-PET was shared with patients for feedback. This progressed into the second draft of the toolkit, which was used as a starting point to gather additional patient and other stakeholder inputs in an iterative process during further development.

##### PABs

The PABs facilitated the identification of patient priorities and highlighted the unique value of including patients in the clinical development process.

##### PAB I (Virtual Meeting)—P-PET

Research related to the P-PET continued with a virtual meeting with 8 patients (7 were members of the previous in-person PAB [*PAB I (First Meeting)*]). Input on a sample SPFQ question was also sought and is described (*PAB I (Virtual Meeting)—SPFQ*).

Patients were shown a sample visual aid of a study schedule and samples of inclusion/exclusion criteria in lay language. The PAB commented on the clarity of the materials and offered improvements to help patients’ understanding of the materials. The PAB also offered suggestions in preparing for the upcoming second in-person meeting (e.g., providing pre-read materials in advance), which were ultimately included in the final P-PET.

##### PAB I (Second In-Person Meeting)—P-PET

Research continued with a second in-person PAB meeting (including 9 patient advisors, most of whom attended the first in-person PAB and the virtual PAB). Input on the SPFQ was also sought (*PAB I (Second In-Person Meeting)—SPFQ*).

The first draft of the P-PET was reviewed with the PAB members. This review included defining selected sections of a protocol, giving an example, and asking questions of the advisors regarding clarity, background, gaps, suggestions, and how experience in clinical studies might be improved. They commented on what sponsors should ask and consider, what patients want to know, and on specific recommendations regarding the presentation/explanation of the various protocol sections. They offered helpful suggestions in conducting a protocol-focused PAB (e.g., providing follow-up back to patient advisors regarding changes made based on feedback, providing definitions of clinical measures). Their feedback was incorporated into the next version of the P-PET used in the Proof-of-Concept testing conducted with patients diagnosed with lupus.

##### PAB II

The P-PET was piloted using a redacted UCB Biosciences, Inc. Phase 3 clinical study protocol for a completed lupus study as a model. A PAB was conducted with 9 patients of diverse backgrounds (age, gender, race, ethnicity, and region [US and Europe]) diagnosed with Systemic Lupus Erythematosus, most of whom had participated in and completed a clinical study in the previous 3 years. The objective was to gain insights by conducting a retrospective simulation on an actual completed protocol to better understand patient burden with respect to study design and inclusion/exclusion criteria, and to assess the execution of the PAB to further refine the User Guide.

##### MC Interviews—P-PET

Members of the PE team conducted additional rounds of SME interviews within their own member companies; the SMEs represented different levels of the organization, study functions, and extents of experience with similar tools. Input on the SPFQ was also sought (*MC Interviews—SPFQ*).

Versions of the tools were shared with SMEs who were interviewed regarding content. Feedback was provided to a third-party, who compiled, de-identified, and aggregated the feedback before dissemination to the PE team.

##### MC Experience Survey

A survey including 32 questions was asked of 51 SMEs across 18 MCs regarding experience, impact, and best practices in the implementation and use of patient engagement activities.

#### Part 2b: SPFQ Development

Concepts from the literature review, the PAB, the SAG, 1:1 patient concept elicitation interviews, and MC interviews (Part 1: Shared Research) were used to identify a conceptual framework for the SPFQ. Development of the initial version of the SPFQ was based on shared best practices and methodology typically used in creating assessment tools for clinical studies.

During development, an initial draft of the SPFQ was evaluated and compared with a similar instrument, the Trial Feedback Questionnaire (TFQ), developed by a MC.

Based on the evaluation, the PE team decided to adopt as its working draft the TFQ, a published tool available for public use. This decision permitted the PE team to focus resources on expanding the tool for global use as many translations of the TFQ were already available or in progress. Work in other countries is ongoing to ensure conceptual confirmation and cultural validation. This work is being done by an external experienced life sciences consulting firm and will be reported in a separate publication.

##### PABs

*PAB I (Virtual Meeting)—SPFQ* Input on a sample SPFQ question was sought and patients provided valuable suggestions as to the clarity of the sample question and the appropriateness of the rating scale.

*PAB I (Second In-Person Meeting) – SPFQ* Research related to the SPFQ continued with a second in-person PAB meeting. A sample study start-up questionnaire and a cognitive debriefing worksheet were completed by the PAB members, followed by group discussion. Input on the P-PET was also sought (PAB I (Second In-Person Meeting) – P-PET).

##### SAG (Virtual Meeting)

An additional virtual SAG Meeting was conducted with 6 members (a subset of those consulted during the shared research [**SAG I**]) to present an overview of the PE Initiative and to obtain feedback on the SPFQ.

The SAG provided feedback in response to both specific and open-ended questions on a sample SPFQ. Comments included thoughts on.

- the value of the SPFQ
- what would facilitate or be barriers to the site administering (or to the participant completing) the SPFQ
- the timing of administration during a clinical study
- how to administer the SPFQ in the most patient-friendly manner
- the most useful format(s) for collection of these data
- general comments and experience with similar instruments, if any.

##### MC Interviews*—*SPFQ

Additional rounds of interviews were conducted with SMEs from MCs who had specific skill sets (e.g., quantitative scientists, outcomes research specialists). SMEs were from different levels of the organization and study functions and had different extents of experience with similar tools. Input on the P-PET was also sought (*MC Interviews—P-PET*).

The first versions of the SPFQ User Guide and the SPFQ were shared and SMEs were interviewed by a PE team member from the SME’s respective company regarding its content. The feedback was provided to a third-party consultant, who compiled, de-identified, and aggregated the feedback before dissemination to the PE team.

### Part 3: US Food and Drug Administration (FDA) Feedback and MC Simulation Testing

#### US FDA Feedback

The PE team met with the representatives from the US FDA in Silver Spring, MD (including representatives from the Office of the Commissioner; The Center for Biologics Evaluation and Research Office of Biostatistics and Epidemiology; The Center for Drug Evaluation and Research including Office of Translational Sciences, Office of Medical Policy, Office of New Drugs, and Office of Scientific Investigations; The Center for Devices and Radiological Health; and the Oncology Center of Excellence). The objective of this meeting was to provide an overview of the PE Initiative draft toolkits and to obtain FDA advice on the toolkit concepts and their alignment to FDA’s patient-focused drug development initiatives.

#### Simulation Testing

Once the pilot versions were finalized, the P-PET and SPFQ went through a final review by MCs. MCs were asked to either provide comments on the tools or to use the tools in a simulated patient feedback exercise for hypothetical feedback.

The objectives were to.

- test the ability of sponsors to voluntarily implement these tools within their organizations,
- assess if the guidance is flexible enough to be used across separate sponsor companies with varying organizational structures, and
- ensure that all key stakeholders involved in the collection of patient experience information had an opportunity to assess the tools and provide critical feedback relevant for successful implementation.

In total, 16 MCs participated in the PE Initiative. Of these, 7 MCs provided simulation feedback on the SPFQ and 7 MCs provided simulation feedback on the P-PET, with some overlap. This feedback was provided to a third-party consultant who compiled, de-identified, and aggregated the information before dissemination to the PE team. MCs then had one final review of the toolkits prior to finalization for public release.

## Results

### Part 1: Shared Research: Literature Review and Stakeholder Research

A review of the literature led to a framework describing the factors affecting patient perceptions of clinical studies. The framework (Fig. 2) was used for initial development of the tools.

Activation/empowerment factors identified from the literature review that affect a patient’s decision to participate in clinical research are summarized:

- *Personal circumstances* Caregiver needs, healthcare access, family situation, intellectual aptitude/degree of health literacy
- *Health beliefs* Personal, familial, community, religious beliefs
- *Health status* Newly diagnosed, genetic status, acute condition, chronic condition, terminal condition
- *Access and affordability* Travel/geography, public relations, time commitment, study availability, advertising, devices, technologies

**Fig. 3:**
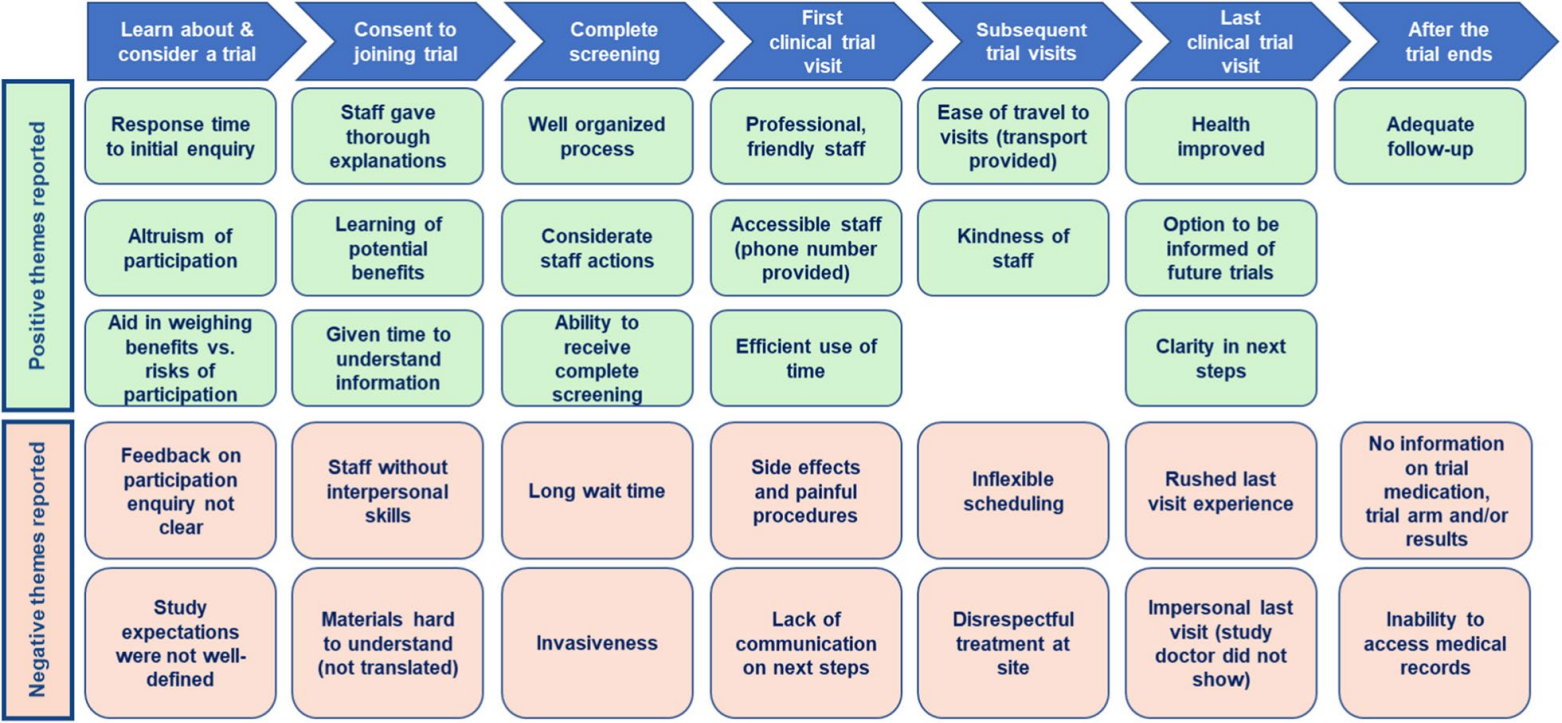
Factors that affect overall study experience

Figure 3 illustrates the findings from PAB 1 which are consistent with information found in the literature review and framework.

The outcome of this collaboration resulted in the first draft of the P-PET.

**Fig. 4:**
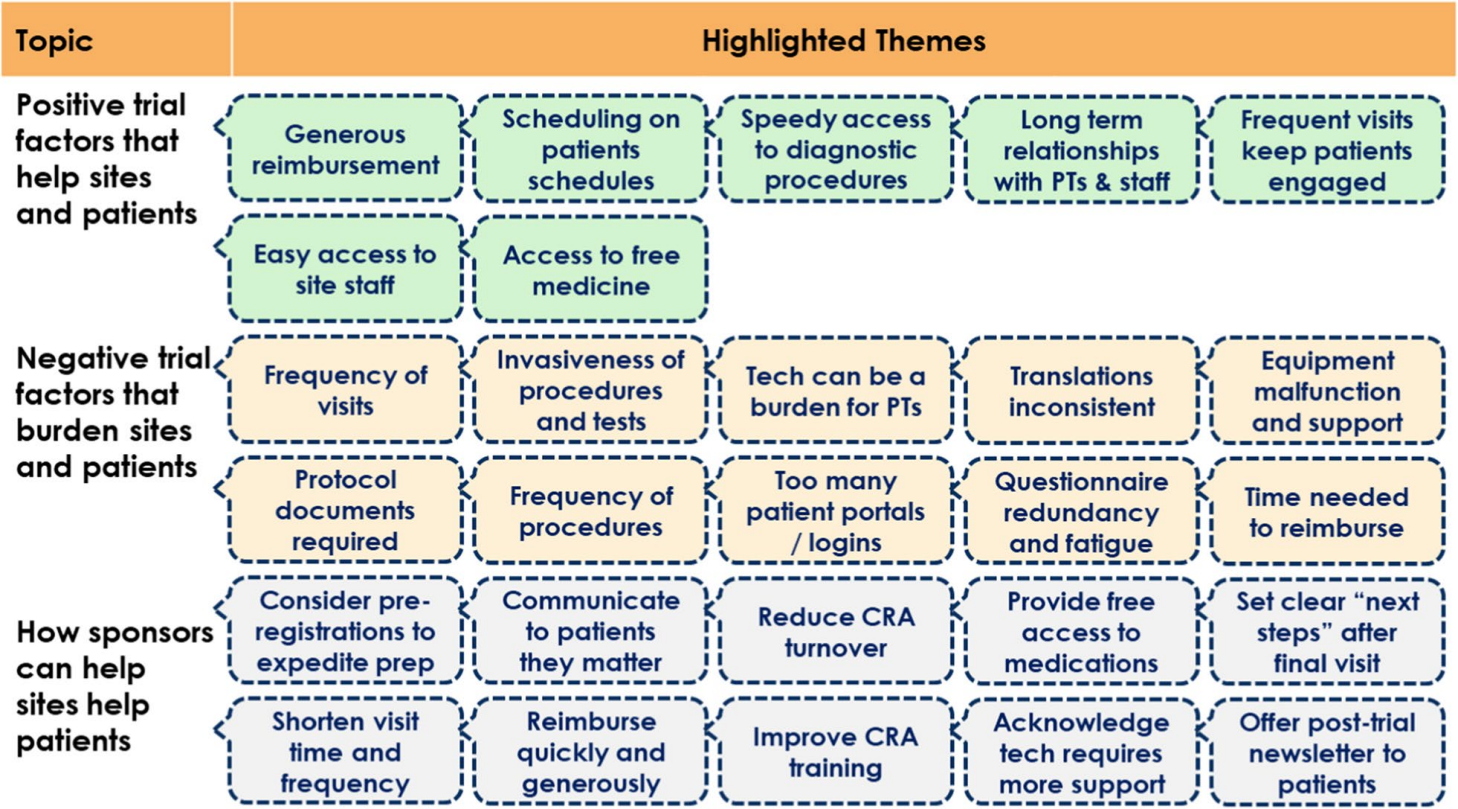
Site advisor-identified factors that affect sites and patients

The three primary themes identified by the SAG regarding patient burden were lengthy, complex or frequent visit schedules; technology/equipment challenges; and quality of study materials/translations (e.g., informed consent). Figure 4 presents several highlighted themes.

**Fig. 5:**
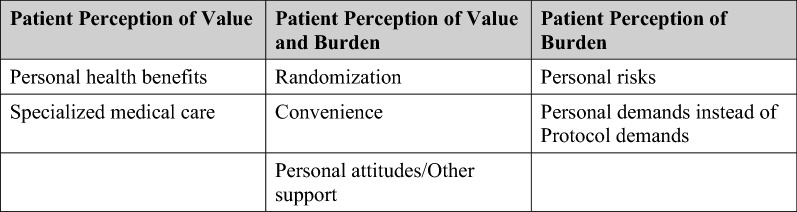
SAG rankings of factors affecting patient perceptions

The SAG ranked factors that affect patient perception of value, perception of both value and burden, and perception of burden as shown in Fig. 5.

Regarding other factors, the SAG ranked patient treatment by site staff and personal characteristics above external factors. The SAG also added factors perceived as “missing” from the framework.

The concepts elicited in patient interviews were similar to those identified in the literature and from the PAB and the SAG. The interviewees also identified other themes perceived as “related” or “missing.”

Finally, interviews with MCs identified factors that.

- had high potential to affect patient willingness to join a clinical study over which sponsors had a high degree of control (e.g., results transparency, convenience, protocol demands),
- were perceived as “missing,” and
- might be organized under different or additional concepts.

The knowledge cultivated during the shared research phase led to a conceptualization of the types of information that may need to be measured to optimize patient experience, and when important patient feedback about study protocols, assessments, and procedures should be obtained to optimize patient experience. The PE team recognized the need to develop tools to gain patient input during protocol design (the P-PET) as well as at various times during a clinical study (the SPFQ).

### Part 2: Development of Draft Toolkits

PAB II, which reviewed the completed UCB protocol as part of a simulation exercise, recommended potential modifications to the protocol, including study design, inclusion/exclusion criteria, risk/benefit, dosing, schedule of assessments and changes to the patient outreach materials. Specific suggestions were incorporated into the P-PET and included pre-read materials; explanation of clinical research terms and medical jargon in plain language; and effective management of patient and researcher interaction. This led to updates to The User Guide and Resource Guide, and P-PET templates were generated, including patient and study team reports, satisfaction surveys, a PAB appreciation letter, and a PAB execution template.

MC interviews covering the P-PET and SPFQ led to further revisions to the draft P-PET. The data gathered helped update the P-PET and the SPFQ User Guide, as well establish a FAQ document.

The PE team determined there were three key time points to request participant feedback during study participation (beginning, during, and end). The SPFQ conceptual framework also yielded content for questionnaire items.

Evaluation of the initial draft of the SPFQ compared with a similar instrument, the Trial Feedback Questionnaire (TFQ), developed by a MC determined that both had the same objectives, were developed using similar methodology, and had an overlap of key domains and themes. The TFQ, already a published tool available for public use, was adopted as the working draft in the place of the then-draft SPFQ.

PAB I (Second In-Person Meeting—SPFQ) provided actionable feedback on the questionnaire: rewording of questions to be more health literate and less complex, adding a neutral response option, increasing clarity with use of lay terms, and format (electronic vs paper), and participation considerations.

SAG (virtual meeting) input was incorporated into the SPFQ and/or User Guide, including

- considerations on timing and administering the SPFQ in the most patient-friendly manner,
- addition of resources for tool administration and a contact number for questions,
- guidance on patient privacy, and
- considerations on mode of survey administration (paper vs. electronic).

Blinded and aggregated feedback from MC interviews (SPFQ) informed revisions to the draft SPFQ including a scaling of response and a reduction in the number of questions to reduce patient burden. The SPFQ User Guide was updated, including changes to the definitions of the SPFQ team (in-house or outsourced), regional regulatory considerations, assessment of the reading level of the SPFQ, and addition of process flowchart/timeline.

### Part 3—FDA and Final MC Feedback

The FDA indicated that the Patient Experience tools provide the starting point of a journey to include patient engagement earlier in the drug development continuum. The FDA described the tools as very workable and as complementary to FDA efforts. The FDA was interested in the measurement/collection of data and in the tangible value the tools are expected to provide (e.g., patient retention, patient insights). The Agency expressed interest in case studies and metrics of using the toolkits, and a desire to collaborate and engage further on the tools.

The FDA was interested to know results that might come out of the pilot. For the SPFQ specifically, the Agency offered the following perspectives:

- Ensure that execution of the SPFQ does not add to patient burden/questionnaire fatigue. (Draft version reviewed by the FDA included 5 to 8 core questions, the revised SPFQ contains 4 to 8 core questions).
- Suggestion to assess how some of the information collected from the SPFQ could potentially be used to inform future study design.

Based on de-identified and aggregated feedback from the P-PET simulation, MCs liked how the tool highlights various components for consideration when engaging patients and most felt the P-PET would be effective in gaining useful feedback. Some MCs were unclear how to utilize the Resource Guide; it was simplified and more instruction on how to use the question bank was added.

Based on de-identified and aggregated feedback from the SPFQ simulation, most MCs indicated that the inclusion of the SPFQ and use of the User Guide fit within current processes in their company. Minor additions and updates were made, including considerations for the timing of administration of the SPFQ and perceived impact of obtaining feedback during a clinical study.

### Part 4: Launch of the Toolkits

The P-PET and SPFQ Toolkit are available free for use on the TransCelerate Patient Experience Initiative Assets website (Appendix 2). These tools can be integrated into operational business processes by all sponsors of clinical research based upon individual organizational needs. Any adoption is voluntary and based solely on the company’s/party’s unilateral decision.

## Discussion

Providing easy strategies for recording patient and study participant feedback in clinical research is part of a comprehensive strategy of patient engagement. Utilizing the toolkits to gather this feedback in a systematic fashion creates the potential to improve the patient experience. The mitigation of identified patient concerns can result in reduced study drop-out, improved adherence, and help with common recruitment and retention challenges. This approach supports the fundamental elements and objectives of patient-focused drug development.

There is potential value in collecting and reviewing data systematically within organizations. Overall patient experiences can be improved by sharing patient input across study teams, clinical programs, and therapeutic areas, as well as in creating use cases for broad stakeholder use and awareness. Another opportunity would be to provide sites with supplemental support through shared best practices and timely feedback, which could strengthen site relationships and positively impact study participant experience.

The first versions of the P-PET and SPFQ toolkit are now available for free from TransCelerate. They represent an opportunity to obtain meaningful patient input on clinical study planning and execution, and to improve patient experience. It is anticipated that their use will identify additional areas for improvement or expansion of content, and therefore future updates and versions of the toolkits.

## Conclusions

By leveraging the P-PET and/or the SPFQ Toolkit, clinical research sponsors and all stakeholders seeking to implement a methodology designed to improve the patient experience potentially could.

- decrease the patient burden of participating in clinical studies,
- positively impact patient adherence and compliance to clinical study procedures,
- increase patient trust and engagement in the clinical development process through improved communication and information-sharing pathways,
- enable clinical protocols to become more patient-centered through a cycle of engaging patients to obtain their insights during protocol development and gaining meaningful feedback on study participation experiences of relevance to them, and
- share meaningful data across studies, programs, and therapeutic areas within organizations to help improve overall patient experiences.

## Appendix 1: MCs, SME Roles, and Therapeutic Areas Represented in MC Feedback

**Table:**

PE team member companies	Abbvie, Allergan, Amgen, Astellas, AstraZeneca, Boehringer Ingelheim, Bristol-Myers Squibb, EMD Serono/Merck Healthcare KGaA, GlaxoSmithKline, Janssen, Lilly, Merck, Novartis, Novo Nordisk, Pfizer, Roche, Sanofi, and UCB Pharma
SME roles	Decision maker for study design (e.g., clinical science/ development leaders) Influencer responsible for study design and conduct (e.g., operational study team, biostatistics, and patient-reported outcomes [PRO] scientists) Strategic driver of patient centricity (e.g., corporate strategy executives, Chief Patient Officers, patient network and advisory leaders) Decision maker for operational plans (e.g., clinical operations leaders) Senior Stakeholder Engagement Manager Vice President Medical Science, Oncology Clinical Operations Lead; Global Program Lead; Innovation Head Individuals who conduct external patient engagement activities (e.g., advocacy groups)
Therapeutic areas	Anti-Infectives, BioMedicines, Bone, Cardiovascular, Central Nervous System, Dermatology, Devices, Diabetes, Endocrinology, Eye Care, Gastroenterology, Internal Medicine, Immunology, Immuno-oncology, Medical Aesthetics, Metabolism, Oncology, Respiratory, Urology, and Virology

## Appendix 2: Links to the Patient Experience Overview and Toolkits

- Overview Page: https://transceleratebiopharmainc.com/initiatives/patient-experience/
- Asset Page: https://transceleratebiopharmainc.com/patientexperience/
- P-PET Page: https://transceleratebiopharmainc.com/patientexperience/patient-protocol-engagement-toolkit/
- SPFQ Page: https://transceleratebiopharmainc.com/patientexperience/study-participant-feedback-questionnaire/
- **Acknowledgements and References = 500 = 2 pp (16)**
- PE Website: https://transceleratebiopharmainc.com/patientexperience/
- PE Video: https://youtu.be/E7NZFlMvyW8
- P-PET: https://transceleratebiopharmainc.com/patientexperience/patient-protocol-engagement-toolkit/
- SPFQ Toolkit: https://transceleratebiopharmainc.com/patientexperience/study-participant-feedback-questionnaire/

